# Mesenchymal stromal cells (MSCs) and colorectal cancer: a troublesome twosome for the anti-tumour immune response?

**DOI:** 10.18632/oncotarget.11354

**Published:** 2016-08-17

**Authors:** Grace O'Malley, Madelon Heijltjes, Aileen M. Houston, Sweta Rani, Thomas Ritter, Laurence J. Egan, Aideen E. Ryan

**Affiliations:** ^1^ Regenerative Medicine Institute (REMEDI), National Centre for Biomedical Engineering Science (NCBES) NUI, Galway, Ireland; ^2^ Discipline of Pharmacology and Therapeutics, Translational Research Facility, National University of Ireland, Galway, Ireland; ^3^ Leiden University Medical Centre, Leiden, The Netherlands; ^4^ Department of Medicine, University College Cork, Ireland

**Keywords:** mesenchymal stromal cells, tumour microenvironment, colorectal cancer, immunosuppression, immunomodulation

## Abstract

The tumour microenvironment (TME) is an important factor in determining the growth and metastasis of colorectal cancer, and can aid tumours by both establishing an immunosuppressive milieu, allowing the tumour avoid immune clearance, and by hampering the efficacy of various therapeutic regimens. The tumour microenvironment is composed of many cell types including tumour, stromal, endothelial and immune cell populations. It is widely accepted that cells present in the TME acquire distinct functional phenotypes that promote tumorigenesis. One such cell type is the mesenchymal stromal cell (MSC). Evidence suggests that MSCs exert effects in the colorectal tumour microenvironment including the promotion of angiogenesis, invasion and metastasis. MSCs immunomodulatory capacity may represent another largely unexplored central feature of MSCs tumour promoting capacity. There is considerable evidence to suggest that MSCs and their secreted factors can influence the innate and adaptive immune responses. MSC-immune cell interactions can skew the proliferation and functional activity of T-cells, dendritic cells, natural killer cells and macrophages, which could favour tumour growth and enable tumours to evade immune cell clearance. A better understanding of the interactions between the malignant cancer cell and stromal components of the TME is key to the development of more specific and efficacious therapies for colorectal cancer. Here, we review and explore MSC- mediated mechanisms of suppressing anti-tumour immune responses in the colon tumour microenvironment. Elucidation of the precise mechanism of immunomodulation exerted by tumour-educated MSCs is critical to inhibiting immunosuppression and immune evasion established by the TME, thus providing an opportunity for targeted and efficacious immunotherapy for colorectal cancer growth and metastasis.

## INTRODUCTION

Worldwide colorectal cancer (CRC) is the third most common cancer diagnosed in men, and the second most common in women [1]. In 2014 in the US alone, an estimated 130,000 people were diagnosed with colorectal cancer, with 50,000 deaths from the disease [2]. Colorectal cancer develops slowly, beginning with adenoma and progressing over several years to carcinoma [3]. Although the stages of colorectal cancer may be defined [4], the origins of the disease are numerous and multifactorial. Microsatellite instability is detected in about 15% of CRC cases, meaning that these tumours have defective DNA mismatch repair. It is thought that these tumours are more immunogenic than their microsatellite stable counterparts due to the generation of large numbers of abnormal peptides [5]. As such, the tumours are characterised by a larger lymphocyte infiltrate and better prognosis for the patient [5, 6]. In many cases, colon cancer is diagnosed as localised disease, however, the majority of deaths in CRC are due to the development of therapy refractory metastatic disease. Tumour growth and metastasis are promoted by factors in the vicinity of the tumour, known as the tumour microenvironment. The “seed and soil” hypothesis proposed as far back as 1889 by Stephen Paget when he noticed that a cancer cell (the “seed”) would only grow if the environment (the “soil”) was suitable [7]. It is now widely accepted that the microenvironment within which a tumour grows is critical to its survival and progression [8].

The colon tumour microenvironment (TME) is composed of many cell types including endothelial cells, immune cells and fibroblasts [9-12]. These constituents influence the survival and growth of the tumour through secretion of factors necessary for angiogenesis, aiding tumour cells in evading apoptosis, or enabling tumours to evade the immune system detection and elimination [10, 13, 14]. More recently, a newer accomplice to the crime has been identified - the mesenchymal stromal cell (MSC). As described recently by Owens, et al, the stromal cell niche in the intestine is comprised of numerous, heterogenous subsets of stromal cells, defined as CD45^-^ Epcam^-^ cells, and includes fibroblasts, myofibroblasts, and MSCs [15]. In healthy intestine, subsets of stromal cells have been defined by their expression of α-SMA, FAPα^+^, CD90^+^, ICAM-1^+^ and gp38^+^ (Podoplanin). Stromal cells in the intestine can display capacity to sense, initiate and respond to extrinsic immunological ques, including pathogens and inflammation [15]. In fitting with their proposed immunological functions, stromal cells are located close to blood vessels and the lymphatic network and are positioned adjacent to the intestinal epithelial cancer cells. Both the high proportion and localisation of stromal cells in the colon suggest that the function of these cells is likely crucial to intestinal homeostasis and cancer. In this article, we review current knowledge of stromal, tumour and immune cell interactions in colon tumours, with a focus on new data that has increased our understanding of the immunological consequences of stromal cell interactions and how they may be manipulated in the context of colorectal cancer.

## MESENCHYMAL STROMAL CELLS (MSCs) AND CANCER-ASSOCIATED FIBROBLASTS (CAFs) - ONE AND THE SAME?

Mesenchymal stromal cells (MSCs) are non-haematopoietic multipotent adult stromal cells which display a fibroblast-like morphology. MSC reside in the bone marrow, but are also found in tissues such as adipose tissue, umbilical cord blood, and dental pulp [16-18], and support homeostasis in healthy tissue during regeneration and wound healing. MSCs are defined *ex vivo* by the following basal cell surface protein expression: CD45^-^ CD31^-^ CD34^-^ CD14^-^ CD11b^-^ DC79α^-^ CD19^-^ MHC-I ^low^ MHC-II ^low^ and CD105^+^, CD90^+^ and CD73^+^, as well as their ability of tri-lineage differentiation capacity i.e. differentiation to osteoblasts, adipocytes or chondrocytes [19]. While MSCs from other species share the characteristics of tissue culture plastic adherence and tri-lineage differentiation, their cell surface characterisation is more complex and varies greatly between species. For example, Peister et al., found mouse MSCs to express varying levels of CD34 and stem cell antigen-1 (Sca-1) [20].

MSCs have potent immunomodulatory capacity and are being investigated as a cellular therapy for use in a broad range of inflammatory diseases, including osteoarthritis, graft-*versus*-host disease (GvHD), and myocardial infarction (MI) [21]. Characteristics that make MSCs attractive as an immunomodulatory therapy include their ability to home to the sites of inflammation and injury and release growth factors or cytokines, to promote healing [22, 23], to dampen inflammation [24] or to differentiate into various types of damaged tissues [25]. The anti-inflammatory properties of MSCs are dependent on the ability of MSCs to respond to their environment and become “activated”. Pro-inflammatory stimuli, including TNF-α, IL-1β, IL-6 or IFN-γ can enhance the immunosuppressive capabilities of MSCs [26, 27]. This enhanced immunosuppressive ability in response to inflammation is obviously attractive in diseases such as GvHD or MI. In the context of the tumour microenvironment, however, in the presence of a high level of pro-inflammatory signalling, these potent immunomodulatory properties can potentially influence the anti-tumour immune response and angiogenesis [28].

**Figure 1:**
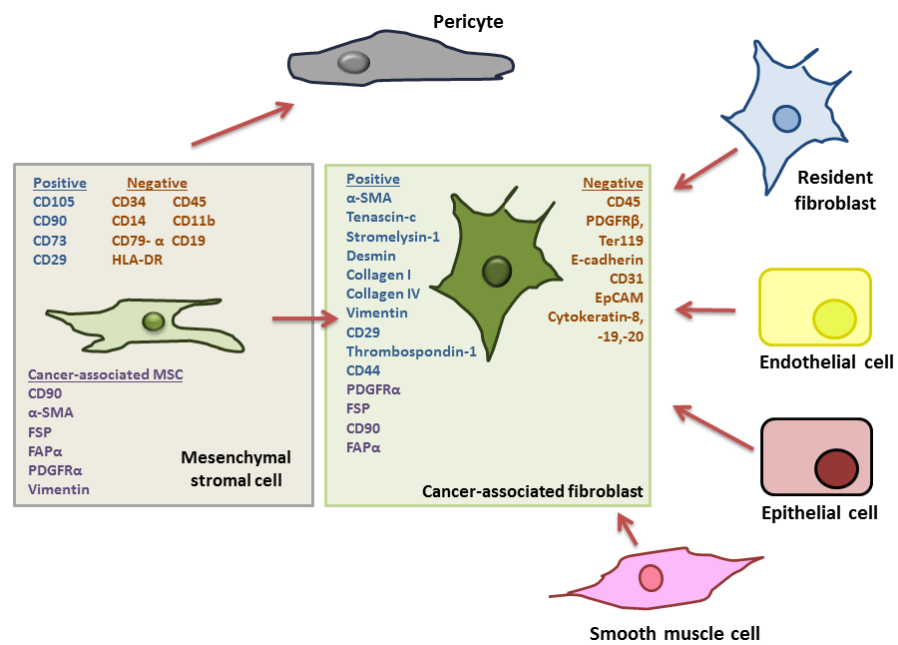
Outline of potential progenitor cells for cancer associated fibroblasts in the tumour microenvironment Adapted from *Cirri et al., (33)*. Cancer-associated fibroblasts (CAFs) represent major and important component of the tumour microenvironment (TME) and have been shown to affect tumour growth and progression. It has been suggested that these CAFs can differentiate from numerous cellular progenitors including tumour-resident fibroblasts, endothelial cells, epithelial cells and smooth muscle cells. However, it has more recently been noted that CAFs share a number of characteristics with mesenchymal stromal cells (MSCs) including the expression of platelet derived growth receptor (PDGFR)-α, and, upon isolation from a tumour MSCs have been shown to express fibroblast-activating protein (FAP) and fibroblast-specific protein (FSP) the reported hallmarks of CAFs. Furthermore, it has been suggested that “CAF” is a cell “state” rather than a specific cell type, pointing to a precursor with a plastic phenotype and robust differentiation capacity, typical of MSCs, For these reasons it has now been hypothesised that MSCs, along with their capacity to differentiate into pericytes, are also the precursors of CAFs in the TME.

The stromal cell compartment of the tumour microenvironment has recently been shown to have important prognostic and diagnostic relevance to patient outcomes in colon cancer. The stromal cell compartment includes cancer associated fibroblasts (CAF), myo-fibroblasts, myeloid cells, endothelial cells and MSCs [8]. Recently, it has been proposed that MSCs are precursors to cancer-associated fibroblasts, and the two cells types have been shown to express similar cell surface markers [29-31]. (Figure 1). Fibroblasts are a population of non-vascular, non-epithelial, non-inflammatory cells that form part of and help synthesise connective tissue [13] They are known to play an active role in wound healing, and the activated fibroblasts found in the tumour microenvironment (CAFs) are believed to be of a similar “wound-heal-promoting” phenotype [13]. Like MSCs, CAFs originate in the bone marrow [29, 32, 33]. In terms of cell surface marker expression, there are many similarities between MSCs and CAFs. MSCs present in tumours have been found to express fibroblast-activating protein (FAP) and fibroblast-specific protein (FSP), CAF-defining markers [31]. Additionally, platelet derived growth factor receptor-alpha (PDGFR)-α has been used to identify CAFs, based on reports demonstrating PDGFR-α expression on up to 90% of stromal fibroblasts in solid tumours [34, 35]. This marker however, is not unique to fibroblasts, and is commonly used to purify murine bone marrow-derived MSCs [36]. The lack of knowledge regarding specific cell surface molecules or cell specific promoters associated with different cell types of mesenchymal origin, including MSCs and CAFs in the tumour stroma, is a major limitation in progressing our understanding of their individual functions in the TME.

In fact, the lack of cell specific markers or cell specific promoters has led to conflicting results in models of colitis associated cancer. To elucidate the role of IKKβ signalling in “intestinal mesenchymal cells” or “CAFs” one study found IKKβ in these cells to be tumour-promoting, the second found it to be anti-tumorigenic [37, 38]. The major difference between these two studies was the use of two different Cre drivers to target the cell in which to delete IKKβ - ColVCre where tumour progression was seen and Col1a2Cre-ER where an anti-tumour effect was observed. In fact many of the markers used to define MSCs, e.g. FSP1 (S100A4) are also expressed by macrophages in the stromal compartment of tumours [39]. The lack of specificity in cell surface markers used to ascribe different populations of stromal cells in the TME has led to much disparity in assigning cellular functions to unique stromal cell populations. The disparity in these studies highlights the heterogeneity that exists in an environment like the intestine and demonstrates the critical importance of defining cellular markers or cell specific promoters that will allow us to confidently identify and target different stromal cell populations in the TME.

As well as sharing cell surface markers with MSCs, CAFs have also been shown to have tumour promoting properties in the TME (Table 1). CAFs have been shown to influence the growth and progression of tumours [30, 40-42]. Activated fibroblasts, or myofibroblasts - identified by their expression of α-smooth muscle actin (α-SMA) - are important forces driving tumour progression not least in the case of colorectal cancer [43, 44]. Conditioned medium from CAFs isolated from patients with metastatic colon cancer promote colon cancer cell proliferation to a greater extent compared to normal fibroblasts from the same organ [40]. MSCs exposed to the conditioned medium from tumours have also been found to exhibit a similar gene expression profile to CAFs [29, 31, 45, 46]. In colorectal cancer, the same effect has been observed i.e. that MSCs injected with CRC cells expressed CAF-defining markers *via* TGF-β/SMAD signalling and promoted tumour growth [45, 46].

**Table 1:** Outline of studies using specific cellular markers to define the role of stromal cells in colon cancer

Study	Cancer type	Source of fibroblasts/ CAFs	CAF marker(s)	Effect on tumour	Effect on immune response
Nakagawa [40]	Metastatic colon cancer	Fibroblasts isolated from 3patients with liver metastasis	Lack of epithelium specific markers cytokeratin-19 and -20. Positive for vimentin (RT-PCR) and α-SMA (immunofluorescence)	Increased HCT116 proliferation *in vivo*	Not assessed
Nagasaki [41]	Colon cancer	Fibroblasts isolated from 64 year old patient	Lack of cytokeratin, positive for CD90 and vimentin (immunostaining) α-SMA (immunofluorescence)	Blocking stromal IL-6 decreased tumour growth and angiogenesis in mouse xenograft model	Not assessed
Zhang [42]	Epithelial Ovarian Carcinoma (EOC)	Fibroblasts isolated from 61 patients with EOC	Negative for cytokeratin-8, positive for FAP and vimentin (immunohistochemistry)	Increased α-SMA staining in advanced disease and in cases with lymph node and omentum metastasis. Positive correlation between α-SMA and lymphatic and microvessel densities *In vitro* fibroblasts promoted invasion and migration of ovarian cancer	Not assessed
Olumi [44]	Prostate cancer	CAFs from 3 prostate cancer patients	Negative for cytokeratin and positive for α-SMA and vimentin (immunofluorescence)	CAFs promoted tumour progression when grafted as tissue recombinants into nude mice. Tissue recombinants + CAFs appeared metastatic, recombinants + normal fibroblasts appeared benign	Not assessed
Direkze [33]	Pancreatic insulinoma	Fibroblasts isolated from mouse bone marrow	α-SMA for myofibroblasts vimentin for fibroblasts (immunostaining)	RIPTag mice administered GFP+ bone marrow via tail vain following whole body irradiation. In pancreatic tumours that developed, 25% of myofibroblats found to be bone marrow derived	Not assessed
Mishra [29]	Breast Cancer	*In vitro* expansion of human bone marrow MSCs.	α-SMA, vimentin, FSA (immunofluorescence)	Tumour-conditioned MSCs co-cultured with tumour cell line increased tumour cell growth and proliferation	Not assessed
Spaeth [31]	Ovarian cancer	Human bone marrow MSCs	FSP, FAP, tenascin-c, thrombospondin-1, stromelysin-1, α-SMA, desmin, VEGF (immunohistochemistry)	Co-injection of MSCs with tumour cells resulted in significantly larger tumours	Not assessed
Erez [34]	Squamous skin carcinoma	CAFs isolated from mouse dysplastic skin	PDGFR- α (flow cytometry)	Tumours co-injected with CAFs demonstrated enhanced growth and vascularisation	Co-injection of CAFs resulted in increased recruitment of macrophages which supported increased tumour vascularisation
Shainagawa [46]	Colon cancer	Human bone marrow MSCs expanded *in vitro*	α-SMA, PDGFR-β, desmin, FSP, FAP (Immunofluorescence)	Tail vein injection of MSCs into tumour bearing mice. MSCs detected in primary tumour site and liver metastasis. Co-injection of MSCs and tumour cells resulted in enhanced tumour growth, increased PCNA-LI, increased MVA and decreased AI	Not assessed
Koliaraki [37]	Colon cancer	Intestinal tissue	CD45, Ter119, CD31 E-cadherin negative, CD29, CD44, CD104- α positive (Flow cytometry). α-SMA, vimentin, collagen IV (immunohistochemistry) Vimentin, collagen IV (flow cytometry)	Deletion of Ikkβ in intestinal mesenchymal cells protected against inflammation associated carcinogenesis	Ikkβ in intestinal mesenchymal cells regulated immune cell infiltrate and cytokine production
Pallangyo [38]	Colon cancer	Intestinal tissue	PDGFRα, CD29, CD44 positive, PDGFRβ CD45, Ter119, CD31 EpCAM negative (flow cytometry) Vimentin, FSP, α-SMA (immunofluorescence)	Lack of Ikkβ in intestinal fibroblasts increases tumour size	Not assessed
Kraman [51]	Lewis lung carcinoma and pancreatic ductal adenocarcinoma	Mouse tumour tissue	FAP, α-SMA, Col I (Immunostaining)		LLC – depletion of FAP^+^ cells induced necrosis of tumour cells. PDA – depletion of FAP+ cells allowed immunogenic control of tumour growth
Feig [52]	Pancreatic ductal adenocarcinoma	Mouse tumour tissue	FAP, α-SMA (Immunostaining) PDGFR- α positive, CD45 negative (Flow cytometry)		Inhibiting CXCR4, a receptor for FAP^+^ stromal cell CXCL12 promoted T cell accumulation and synergised with checkpoint antagonists resulting in tumour regression
Calon [49]	Colon cancer	Human colon adenoma and carcinoma tissue	FAP	Pharmacological inhibition of stromal cell TGF-β signalling blocked initiation of metastasis	Not assessed

A recent review by Madar *et al.*, put forward the notion that “CAF” is a cell “state” rather than a cell type [47], and this fits with the hypothesis that MSCs are CAF-precursors. MSCs are a highly plastic cell type with robust differentiation capacity, are recruited to sites of inflammation and have a functional phenotype that is differentially regulated by inflammatory cytokines [48]. Furthermore, Calon *et al.* found TGF-β levels to be a robust predictor of disease relapse in a cohort of 345 CRC patients. This was an interesting finding in light of studies identifying TGF-β as an important factor in causing the differentiation of MSCs to a CAF-like phenotype [49]. They also engineered CRC cell lines, which display a mutational inactivation of the TGF-β pathway, to secrete active TGF-β1. When injected *in vivo* these cells increased tumour formation compared with controls, and since the tumour cells were insensitive to TGF-β signalling, they concluded that the observed effects were due to TGF-β influencing stromal cells [49].

MSCs also have potent immunosuppressive capabilities and data shows that this property is maintained even as mesenchymal cells differentiate [50]. CAFs, the product of tumour-induced MSC differentiation, have been found to be similarly immunosuppressive. Kraman *et al.*, found that depleting fibroblast-activating protein-α (FAP)^+^ cells in a murine model caused hypoxic necrosis and decreased tumour volumes [51]. This could be reversed by anti-TNF-α or anti-IFN-γ therapy, suggesting that FAP^+^ cells attenuate cellular responses to these cytokines, thus protecting the tumour from cytokine-induced clearance. Similarly, Feig *et al.*, observed reduced tumour growth upon depletion of CAFs (FAP^+^ cells) from a model of pancreatic ductal adenocarcinoma (PDA), but interestingly only in the presence of CD4 and CD8 cells [52]. This effect was mimicked by administration of an inhibitor of CXCR4, the ligand for which, CXCL12, is present in CAFs and was suggested as being responsible for the tumour promoting effects of the CAFs. A rapid accumulation of T-cells in the tumour was observed when CXCR4 was inhibited, thus restoring the ability of the immune system to eliminate the cancer cells. These findings highlight the fact that a better understanding of the precise interactions between tumour cells, stromal cells and immune cell populations that regulate immune responses will enable new strategies for enhancing anti-tumour immunity.

While the limitations in defining exclusive cellular populations of mesenchymal origin has important implications in targeting these defined cell type's *in vivo*, identification of common mechanisms of tumour promotion or immune modulation may represent an alternative approach to understanding their individual cellular functions in the TME. In fact, identification of signalling pathways activated in MSCs in response to inflammatory stimuli which lead to potentiation of their immunosuppressive capacity or even their differentiation to a CAF-like lineage could prove essential for therapeutic targeting approaches. To date, however, limited data exists that propose common signalling pathways that may be relevant to stromal cell immunomodulatory potential in tumours. A limited number of studies have attempted to address this question particularly in relation to harnessing the immunosuppressive potential of MSCs for cellular therapy of inflammatory diseases. NF-κB, PI3K (Phosphatidylinositol-4,5-bisphosphate 3-kinase) and STAT-1 activation have been identified as important signalling pathways that enhance the immunosuppressive capacity of stromal cells [53, 54], however no studies have addressed this in the context of tumours. This highlights that further work is necessary to better understand the mechanisms that control stromal cell mediated immunosuppression in the TME with a view to manipulating these pathways for therapeutic benefit in cancer.

## RECRUITMENT OF MSCs TO THE TUMOUR MICROENVIRONMENT

Inflammation is the body's response to tissue damage or injury [55, 56]. There is a strong and well established link between chronic inflammation and the development of colorectal cancer, and this has led many researchers to compare the TME to a “wound that does not heal” [57, 58]. In this sense, the factors that orchestrate recruitment of cells to the TME are likely to be similar to that of a wound, to counteract inflammation and promote wound healing. While desirable in a wound, this type of response is unfavourable in CRC and undoubtedly aids to progression of the disease.

Research on the molecular pathways associated with inflammation associated CRC has identified colon epithelial cell NF-κB, STAT3 and STAT6 activation with the progression of chronic intestinal inflammation to overt CRC [58-61]. In colorectal cancer, NF-κB has been described as the “critical link” between inflammation and cancer, and also found to feature in the wound healing process [58, 62, 63]. Research focusing on the recruitment of MSCs to tumour cells has identified the importance of various signalling molecules in this process, including CXCR4, MCP-1 and VCAM-1, all of which are regulated by NF-κB [64-72]. Shi *et al.*, found the CXCR4/SDF1 signalling axis to be of importance in MSC homing to the bone marrow in irradiated NOD/SCID mice [68]. This was confirmed by Gao *et al.*, who identified SDF1 to be an important stimulus for MSCs to migrate towards tumour conditioned medium [69]. Other important factors implicated in this process are MCP-1, the blockade of which significantly reduced the migration of MSCs to breast tumour xenografts in mice, and VCAM-1 which has been shown to enhance MSC migration to glioma cells *in vitro* [70, 71]. With regards specifically to the setting of colorectal cancer, a study by Uchibori *et al.*, in 2013 confirmed an important role for VCAM-1 in the process of migration [72]. In addition to this important role for tumour cell NF-κB, recent research has shown that stromal cell NF-κB is also has a part to play in colorectal tumourigenesis, when a decreased tumour incidence was identified following intestinal stromal cell specific deletion of IKKβ [37]. In the context of inflammation associated cancer and spontaneous CRC, it is likely that there are multiple mechanisms involved in MSC recruitment to the TME. It is well established that MSCs are recruited to the tumour microenvironment and once there, act to alter tumour biology. In depth knowledge of how tumours regulate the process of MSC recruitment and how MSC migration is affected by inflammation in the tumour microenvironment is needed. More specifically, identification of tumour specific mechanisms that regulate MSC migration, and an understanding of how these mechanisms can be targeted is essential.

## MSCs PROMOTE TUMOURIGENESIS IN VIVO

Apart from their proposed role as CAF precursors, MSCs have also been shown to be capable of modulating colon cancer cell activity through other mechanisms. In fact MSCs in CRC have been shown to directly influence at least three of the six seminal “Hallmarks of cancer” proposed by Hanahan and Weinberg [73], namely evasion of apoptosis [46], sustained angiogenesis [46, 74, 75] and tissue invasion and metastasis [46, 75, 76].

Numerous mechanisms are responsible for the observed effects of MSCs on colon tumour activity. Hogan *et al.*, found bone marrow derived MSC (BM-MSC)-secreted PAI-1 to be responsible for the increases they saw in HCT116 and HT29 colon tumour cell migration *in vitro* [76]. Liu *et al.*, found pre-treating BM-MSCs with inflammatory cytokines induced VEGF expression *via* HIF-1α signalling in MSCs and resulted in increased angiogenesis observed in tumours following C26 colon cancer cell and MSC co-injection [74]. Lin *et al.*, showed that MSC-secreted IL-6 and Notch1 and CD44 induction in HCT116 increased metastatic potential in tumours formed following co-injection of HCT116 and colon cancer-MSCs in Balb/c nu mice [77]. De Boeck *et al.*, found that BM-MSCs increased the invasion, survival and tumorigenesis of various colon cancer cell lines *in vitro* through the release of soluble NRG1 and subsequent activation of the HER2/HER3-dependent PI3K/Akt signalling cascade in colon tumour cells, and that co-administration of these BM-MSCs with the various cancer cell lines *in vivo* increased the percentage of animals presenting with tumours a number of weeks after injection [75]. Similarly, Huang *et al.*, found that MSCs enhanced angiogenesis and migration of tumours formed in athymic nude Balb/c mice following administration of HT29 colon cancer cells, and identified IL-6 secretion by MSCs as the putative mechanism responsible for these increases [78].

**Figure 2:**
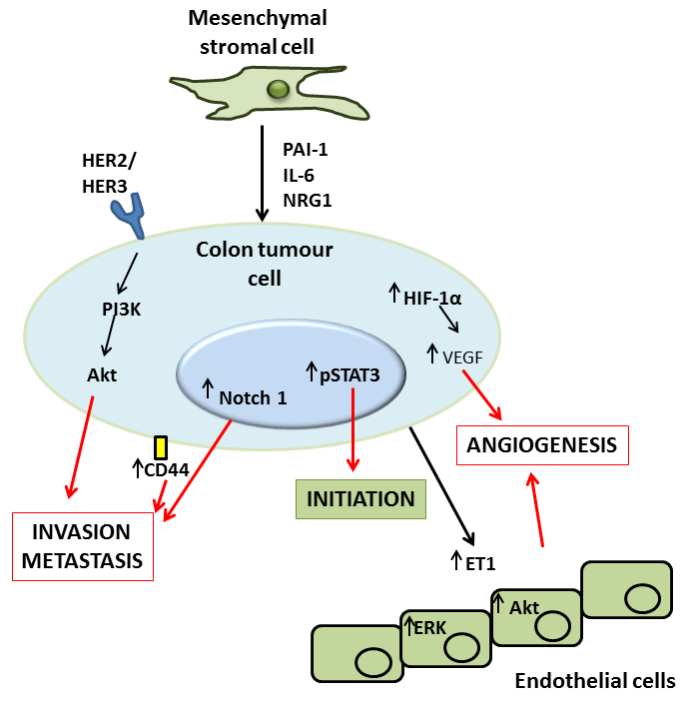
Molecular mechanisms of MSC mediated induction of colon tumour cell initiation, angiogenesis, invasion and metastasis MSCs have been demonstrated to exert direct effects upon tumour cells *via* secretion of factors like plasminogen activator inhibitor (PAI)-1, interleukin (IL)-6 and neuregulin (NRG)1, or by activation of the human epidermal growth factor receptor (HER)2/3 receptor. The result of this signalling is activation of a number of pathways in the tumour, the net result of which is tumour promotion. Activation of HER2/3 leads to an increased invasive and metastatic capacity in the tumour *via* the phosphatidylinositol-4,5-bisphosphate 3-kinase (PI3K) and Akt signalling pathway. A similar effect on invasion and metastasis results from MSC-induced increases in Notch1 and CD44 signalling in colon tumour cells. Additionally, MSCs can induce phospho-signal transducer and activator of transcription (STAT)3 signalling in tumour cells which has been described as important in the initiation of tumourigenesis. Finally MSC induced increases in hypoxia-inducible factor (HIF)-1α, vascular endothelial growth factor (VEGF) and endothelin (ET)-1 can either directly, or indirectly *via* actions by tumour cells upon healthy endothelial cells, induce tumour angiogenesis, another important factor in increasing the growth and progression of tumours both by providing a means for the tumour to travel to distant sites, and delivering nutrients essential for the survival of the tumour.

Accumulating evidence suggests that MSCs enhance various aspects of colon tumour cell biology which favours tumour growth, survival and progression (Figure 2). While this may be the case, the studies referenced here are not without limitations. Firstly, as was noted in the review by Hogan *et al.*, there is a lack of uniformity in much of this work, particularly with regard to the variation in the numbers of cells and ratios of MSCs to tumour cells being administered in each study, and the physiological relevance of the tumour cell:MSC ratios [79]. The physiologically relevant ratio of MSCs present in the colorectal cancer microenvironment is likely to be crucial in fully assessing the specific role MSCs play in the CRC TME and indeed other cancers. Secondly, the majority of the preclinical studies in this area rely on the use of xenograft models of cancer, namely human colon cancer cells, often HT29 or HCT116, being co-administered with human MSCs, bone marrow-derived or otherwise, to a mouse lacking a fully competent immune system, commonly athymic nude Balb/c or Swiss nu/nu mice. While this model has its advantages and is clearly necessary to facilitate the engraftment and outgrowth of the administered tumour cells that could otherwise be rejected by the host animal, the autologous nature of the disease is not accurately represented, and aspects of the “anti-tumour-immunity machinery” are absent. These immune cell components such as T-cells, macrophages, dendritic cells and natural killer cells could, in fact, be central players in MSC-mediated tumourigenesis. The ability to reliably identify the location and ratio of tumour MSCs would facilitate approaches to comprehensively investigate their influence on tumour cells and immune cells in both physiologically relevant models and human specimens.

## MSCs AS MEDIATORS OF ANTI-TUMOUR IMMUNITY

**Figure 3:**
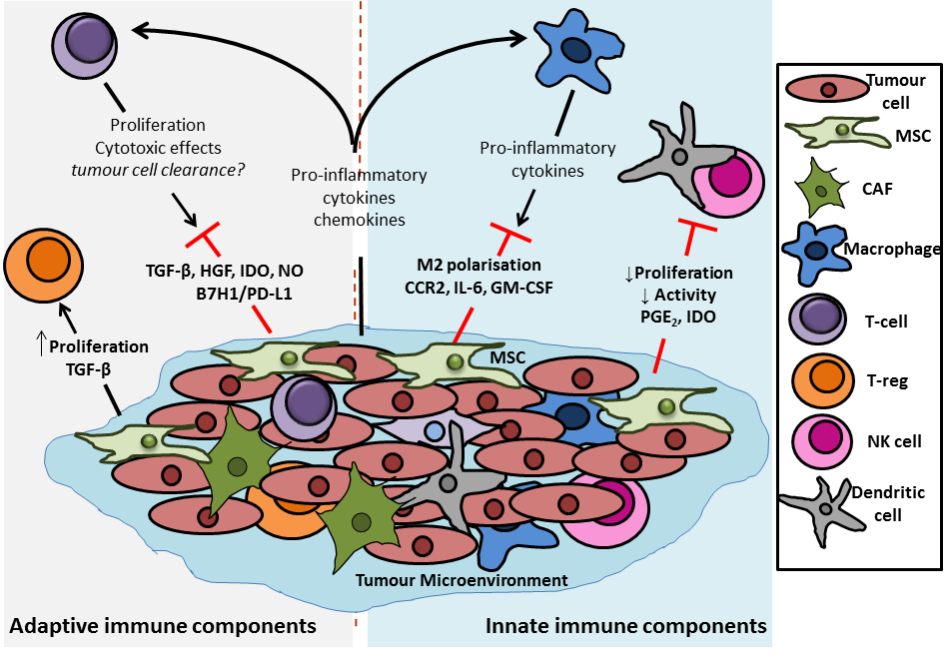
Outline of the potential immunomodulatory effects of MSCs in the colon tumour microenvironment MSCs have been shown to have potent immunomodulatory effects, acting on components of both the innate and adaptive immune system. In terms of innate immunity it has been shown that MSCs can dampen any early immune response that the host system may mount against a transformed tumour cell. This dampening of the initial response is due to the ability of the MSCs to decrease the proliferation and activation of dendritic cells (DCs) and natural killer (NK) cells, the potential “first responders” of an anti-tumour immune response. Macrophages represent another important group of innate immune cells with the potential to exert anti-tumour response, particularly *via* release of pro-inflammatory cytokines from their M1, pro-inflammatory-like phenotype. This effect is also hampered by MSCs which act to induce a more M2, anti-inflammatory phenotype in macrophages, thus inhibiting their capacity to clear transformed cells. With regards to the adaptive immune compartment, it has been demonstrated that MSCs have differential effects upon different t-cell populations. Evidence from the literature shows that release of factors such as transforming growth factor (TGF)-β, hepatocyte growth factor (HGF), indoleamine 2,3-dioxygenase (IDO) (human), nand nitric oxide (NO) (rodent) from, and induction of B7H1 or PD-L1 expression on MSCs directly reduces the proliferation and cytotoxic effects of effector CD4^+^ and CD8^+^ T cells, thus inhibiting their tumour-clearing capacity. In contrast, signalling by MSCs has been reported to increase the proliferation of regulatory t cells (T_regs_), a population which act to suppress the activity of other effector t cells. In healthy tissue this supressed auto-immunity and tolerance of “self” is desirable, but in a tumour, where the aim is to clear transformed cells, the increased proliferation of T_regs_ can be detrimental to tumour lysing and clearing by cytotoxic T cells.

The theory of cancer immunoediting describes the ability of the immune system to survey the landscape for potential cancerous growths, and to interact with tumours and directly alter their immunogenicity [80, 81]. According to this theory, the immune system can protect the host by recognising immunogenic tumour cells and mounting a T-cell mediated response to actively eliminate these cells (the “elimination” phase) [82, 83]. The corollary of this elimination of immunogenic cells is that the outgrowth of other, less immunogenic tumour cells may be favoured (the “equilibrium” and “escape” phases) [81, 82, 84]. There are undoubtedly numerous mechanisms by which tumour cells interact with and avoid clearance by the immune system such as insensitivity to interferon (IFN)-γ, and alteration in tumour cell MHC class I molecules and antigen presentation [85-87]. However, evidence now suggests that MSCs may have an important role to play in this process. MSCs possess immune regulatory functions, and in particular have been shown to be immunosuppressive in response to pro-inflammatory cytokines or TLR ligation [88-91]. In the colorectal TME, where inflammatory signalling is prevalent, it has been suggested that MSCs interact with immune cells, resulting in dampening of anti-tumour responses and thereby promoting tumorigenesis [92-94]. Numerous mechanisms have implicated in how MSCs modulate the immune components of the TME. Quite often the focus has been on MSC-T-cell interactions, but influences on other immune cell subpopulations such as macrophages and dendritic cells (DCs) have also been identified. In the context of the TME, MSC influences on the phenotype of these immune cells will dramatically affect tumour progression and a thus a better understanding of the precise mechanisms involved is critical to the development of more efficacious immunotherapies (Figure 3).

## INFLUENCE OF MSCs ON THE INNATE IMMUNE SYSTEM IN THE TUMOUR MICROENVIRONMENT

As noted by Lamagna *et al.*, macrophages could be considered the first line of defence against tumours given that they colonise rapidly and secrete cytokines to activate other innate immune components such as dendritic cells (DCs) and natural killer cells (NK cells) [95]. Additionally, macrophages have been shown to be capable of phagocytosis of dead tumour cells and cross-presention of tumour antigen to CD8+ T cells [96]

### MSC-induced polarisation of macrophages towards an M2 phenotype

Evidence shows that MSCs can exert their tumour promoting effects by interacting with macrophages in the TME [97, 98]. Macrophages can be polarised to an M1 phenotype which secrete reactive oxygen and nitrogen species and inflammatory cytokines, or an M2 phenotype which are involved in the suppression of inflammation and tissue remodelling [99]. Tumour-associated macrophages (TAMs) have been found to be of the M2 phenotype which, in the context of the tumour microenvironment, results in dampening of anti-tumour immune responses [97, 98]. It has been shown that MSCs can polarise macrophages towards this “anti-inflammatory” M2 phenotype, characterised by IL-10 production and decreased iNOS and IL-12 expression [60, 100], and this likely represents another important mechanism by which MSCs aid tumours in evading immune clearance. Ren *et al.*, showed that tumour-educated MSCs greatly enhanced tumour growth, and that this enhancement could be ameliorated by monocyte/macrophage depletion [101]. They also showed that these tumour-educated MSCs expressed high levels of CCR2, the major cytokine involved in monocyte chemotaxis [102]. Although these studies demonstrate the ability of MSCs to induce and M2 phenotype, the factors responsible for this induction remain elusive. Factors implicated in this switching include soluble mediators such as CCR2, IL-6 and GM-CSF [101, 103]. M2-like macrophages could be important targets in the context of developing novel therapeutics or adjuncts to improve the efficacy of those currently used [60, 97, 104]. However, macrophages perform several essential functions throughout the body and so caution must be taken in therapeutically targeting such a cell. Identification of factors released by MSCs in the tumour microenvironment that are responsible for inducing an M2 macrophage phenotype could be even more advantageous for the development of cancer immunotherapy, the effect of which is largely dependent on macrophage effector functions, including antibody dependent cellular cytotoxicity and phagocytosis.

### MSCs induce suppression and altered function in dendritic cells, natural killer cells, neutrophils and myeloid derived suppressor cells

Although most studies looking at the importance of the immune cell components to tumour growth focus elsewhere, there is a body of evidence to support the hypothesis that dendritic cells (DCs) and natural killer (NK) cells also have an important role to play in the anti-tumour immune response [105-107]. Furthermore, DCs and NK cells are also known important components of the colorectal tumour microenvironment [28]. Tumour cell antigens, released either in the form of dying tumour cells or soluble antigen, can be endocytosed by dendritic cells in the TME. Dendritic cells undergo maturation and migrate to secondary lymphoid organs where they can present processed tumor antigens as peptides bound to class I and II MHC molecules to prime effector CD8+ and helper CD4+ T cells, respectively [108]

Despite the lack of studies related directly to the effects of MSCs on DCs and NK cell effector functions in the tumour microenvironment, it is likely their effects are similar to those described in other tissues. Aggarwal *et al.*, showed using co-cultures that MSCs can suppress both proliferation and cytokine secretion, not only by T-cells, but also by DCs, and NK cells, and that these effects can be reversed by PGE_2_ synthesis inhibitors, suggesting PGE_2_ may be also be important in MSC-induced immunosuppression [109]. This is a potentially important finding in the context of CRC. Non-steroidal anti-inflammatory drugs (NSAIDS) have been shown to have a protective effect against the development of CRC [110-112] owing to their ability to inhibit cyclooxygenase (COX)-2, the enzyme that is responsible for the production of PGE_2_ from arachidonic acid [113, 114]. Spaggiari *et al.*, showed that MSCs inhibited both the proliferation and effector functions of NK cells, and the maturation and effector functions of DCs, and that these effects were, as in the case of T-cells, mediated by IDO and PGE_2_, molecules released by MSCs in response to inflammatory signals that are prevalent in the colon TME [115, 116].

Myeloid-derived-suppressor cells (MDSCs) are another family of immune cells derived from myeloid progenitors and shown in humans to express varying levels of CD11b, CD14, CD15 and CD33 [117, 118]. MDSCs have an immature phenotype and the potential to suppress T cell responses *via* expression of arginase 1, inducible NOS, TGF-β, IL-10, COX2 or the induction of T_regs_ [117, 119]. Research has shown that these MDSCs can affect tumour growth and progression in a number of ways, including protecting the tumour from immune mediated clearance by their secretion of NOS, TGF-β, IL-10, PGE2 [118, 119]

Factors responsible for the accumulation of MDSCs in the tumour microenvironment include GM-CSF, G-CSF, M-CSF, all of which are produced by MSCs, suggesting the potential role of MSCs in recruitment and accumulation of MDSCs in the tumour [118, 119]

Evidence suggests that tumour-derived MSCs have an even more pronounced effect on MDSCs, as demonstrated by the induced expansion of MDSCs *in vitro* when in co-culture with tumour derived MSCs. Furthermore, these expanded MDSCs were functionally active and shown to suppress allogeneic T cell proliferation, demonstrating the potential to hamper an anti-tumour immune response [118, 120, 121].

Finally, a limited amount of data exists demonstrating a detrimental role for neutrophils in the TME in terms of promoting tumorigenesis and inhibiting apoptosis [122, 123]. Neutrophils normally play an important role in killing invading microorganisms and although scant, evidence suggests that MSCs have the capacity to interact with this cell type. It has in fact been shown that MSCs can protect neutrophils from apoptosis and that neutrophils activated by tumour-resident MSCs can promote the differentiation of normal MSCs into CAFs, thus contributing to tumour promoting role [124-126]

This data demonstrates that MSCs in the TME have the capacity to suppress effector functions of numerous innate immune cells which act as a first-line defence in detecting transformed cells. Furthermore, this appears to be orchestrated *via* release of soluble factors, namely IDO and PGE_2_ by the MSCs, meaning that these effects can be functional even in the absence of cell contact with MSCs. Elucidation of these complex interactions is vital in order to re-activate the host innate immune system to allow the generation of an effective anti-tumour immune response.

## INFLUENCE OF MCSs ON COMPONENTS OF THE ADAPTIVE IMMUNE SYSTEM

### MSC-mediated effects on T-cells

Much of the evidence for MSC-mediated immunomodulation has focused on the effects of MSCs on the proliferation and/or effector functions of T-cells. Di Nicola *et al.*, found that MSCs could inhibit the proliferation of T cells that had been stimulated by allogeneic peripheral blood lymphocytes (PBLs), dendritic cells (DCs) or phytohemagglutinin (PHA), and that this inhibition was contact-independent and could be reversed using antibodies against TGF-β1 and HGF, suggesting an important role for these cytokines in MSC mediated immunomodulation [89]. Similar work by Meisel *et al.*, identified indoleamine 2,3-dioxygenase (IDO) as important in MSC-mediated T-cell suppression, a finding which has been backed up by other separate studies [127, 128]. These findings are complicated by the fact that the results are only applicable to MSCs isolated from humans and monkeys [129]. When looking at rodent MSCs, IDO appears dispensable, with nitric oxide (NO) found to be responsible for T-cell suppression in this setting [88, 129]. More recently, another, IDO-independent mechanism for MSC-mediated immunosuppression has been identified. Chinnadurai *et al.*, found that while blocking IDO negated the suppressive effect of MSCs on T-cell proliferation, it did not reverse the suppressive effects of MSCs on T-cell function as measured by IFN-γ secretion. Instead, the B7H1 (PD-L1) and B7DC (PD-L2) /PD1 pathways was implicated in the ability of MSCs to suppress T-cell function [130].

While there are clearly numerous suggested mechanisms responsible for this suppressive effect, there is definitive evidence to show that MSCs inhibit T-cell proliferation, but only following T-cell activation, proliferation, and subsequent cytokine production, in particular IFN-γ [88, 128]. T-cell stimulation is a common feature of an inflammatory TME [131]. Furthermore, studies indicate that MSCs are more immunosuppressive when pre-treated with inflammatory cytokines, and can home to sites of inflammation, such as that of the TME [132, 133]. This implies a role for MSCs in migrating to the TME [134] and aiding tumours avoid clearance by interacting with and modulating the host anti-tumour immunity.

Djouad *et al.*, provided some of the earliest evidence to link this MSC-induced immunosuppression to enhanced tumour growth [135]. They found the murine C3 MSCs cell line to be immunosuppressive *in vitro,* and when injected with B16 melanoma cells in allogeneic CH3 mice, these MSCs induced greater tumour formation compared to the control groups of MSCs or B16 cells alone. This result was observed regardless of whether the MSCs and B16 cells were co-injected or injected at separate sites, and for numerous ratios of MSC:B16 up to 1:100, which is important in the context of physiological relevance.

More mechanistic insights came from Patel *et al.*, who found that MSCs protected breast cancer cells from immune clearance by increasing TGF-β production which caused an upregulation in the production of T_regs_ cells [92]. Ljujic *et al.*, found a similar upregulation of T_regs_ along with a decrease in the cytotoxic capacity of CD8^+^ T-cells and NK cells in mice that had been co-administered 4T1 mammary carcinoma cells and hMSCs, and these effects coincided with an increase in tumour growth and metastasis. Mechanistically it was found that the administration of hMSCs caused significant increases in serum levels of TGF-β, IL-4 and IL-10 and a reduced level of IFN-γ in tumour bearing mice, findings which are consistent with MSCs affecting NK and T-cell activity and polarising T-cells away from a Th1 phenotype [92, 136]. Han *et al.*, showed that co-injection of MSCs with B16 cells increased tumour growth over that of B16 cells alone, and that a further increase in growth occurred when the MSCs were pre-treated with IFN-γ and TNF-α. NO production and T-cell suppression was the mechanism cited here as being responsible for the increased growth of tumour cells, owing to the observation that iNOS inhibition reversed the MSC-mediated enhanced tumour growth. As mentioned, however, where MSC NO mediates an immunosuppressive effect in rodents, IDO appears to be the human equivalent [129, 132]. This discrepancy was overcome in a set of elegant experiments by Ling *et al.,* who “humanised” murine MSCs by transfecting iNOS^−/−^ mouse MSCs with the human IDO gene, leading to constitutive IDO expression in these cells [94]. In addition, they engineered the cells to inducibly express human IDO expression under the control of the promoter of the mouse iNOS gene, thus more accurately resembling the human *in vivo* situation whereby IDO expression is upregulated in response to inflammatory signalling. This study concluded that IDO produced by MSCs was responsible for the enhanced tumour growth and that suppression of CD8+ cells was critical for this effect [94]. Effector T-cells have a central role in orchestrating the anti-tumour immune response [137]. Identification of tumour induced mechanisms of MSC-mediated immunomodulation that inhibit T-cell proliferation, differentiation and function will enable the development of more targeted and efficacious immunotherapies, thus reducing reliance on cytotoxic chemotherapeutics and leading to more favourable outcomes for patients.

In the context of colon cancer Liu *et al.*, in 2011, treated murine MSCs with IFN-γ and/or TNF-α and co-injected these cells with murine C26 colon cancer cells into a Balb/c mouse model [74]. Although this study focused primarily on the pro-angiogenic role of these MSCs, it could be that these cells were also exerting suppressive effects on the immune system. This model, unlike many of the other cited here, is a syngeneic model where all cells used are of Balb/c origin, and so is particularly suitable for studying tumour-immune interactions. The authors found that cytokine treated MSCs were more potent promoters of angiogenesis, and attributed this effect to increased VEGF expression in MSCs as a result of HIF-1α signalling. While obviously angiogenic, VEGF has more recently been shown to be immunosuppressive, and so it could be that the MSCs administered in this study are having more than one effect on the tumour cells and actually aiding their avoidance of immune clearance as well as promoting angiogenesis [138, 139]. Another role for VEGF in tumour progression has also been noted - namely macrophage recruitment to the tumour microenvironment and the subsequent development of an immunosuppressive tumour-associated macrophage (TAM) phenotype [139].

What all this data demonstrates is that MSCs play a central role in maintaining homeostasis by modulating the function of innate and adaptive immune cells. Furthermore, it has been shown that MSCs can behave as ‘non-professional antigen presenting cells' as demonstrated by their ability to sense bacteria *via* TLRs/NLRs, their expression of MHC class II and their aforementioned ability to alter T cell function [15, 140]. Evidence in support of this includes, IFN-γ induction of MHC-I and MHC-II expression and the ability to present soluble exogenous antigen leading to the activation or anergy of CD8^+^ and CD4^+^ T cells. [141-143]. Furthermore, CD90^+^ intestinal stromal cells have been shown to be capable of bacteria uptake and phagocytosis [144]. This has provoked the question of whether MSCs are immune cells. A recent review by Hoogduijn addresses this question in detail [50], with the conclusion that MSCs, though immunomodulatory, are not in fact immune cells. In spite of this, the data presented does highlight some interesting idiosyncrasies related to MSC behaviour that does suggest characteristic “immune-like” functions, although to a different extent than professional immune cells. Hoogduijn points out that an immune cell is classified as a cell that protects the host against pathogens and removes debris or diseased cells. To date, data demonstrating these functions in MSC is limited or even absent. At present, in the tumour microenvironment, MSCs act as the “sensors and switchers of inflammation” described by Bernardo and Fibbe in the sense that they detect altered immune activity, respond to it, and as outlined above, interact with and influence the behaviour of components of the innate and adaptive immune system [24], This is an attempt, presumably, to protect the host from excessive inflammation rather than a pathogenic threat. However, little is known about the other immune cell-like functions of MSCs, and even less so the influence of tumour conditioning on these processes. Can MSCs phagocytose transformed or apoptotic tumour cells? Can they induce tolerance by presenting tumour antigen as “self”? Is it possible even that MSCs could be polarised by tumour conditioning to a pro- or anti-tumorigenic phenotype, as is the case for M1/M2 type macrophages. These questions require much investigation before we can fully understand the immunomodulatory capacity of MSCs in maintaining tissue homeostasis and more importantly, their role in dictating the anti-tumour immune response in the colon cancer microenvironment.

## EMERGING CONCEPTS - COULD EXOSOMES PROVIDE A KEY MISSING LINK?

While evidence varies as to the precise mechanisms, it is clear that the interactions between MSCs and components of the TME have profound effects on tumour growth and metastasis. Data suggests that these effects are not dependent on direct contact between tumour cells and MSCs or between MSCs and immune cells. Several MSC derived soluble mediators have been proposed as crirical for this pro-tumourigenic effect, but there is not yet a consensus as to which factors are key players, which play supporting roles, and which ones are dispensable. It seems likely that tumour microenvironment MSC mediated effects require the concerted actions of numerous secreted factors, each with its own important role. The idea that multiple signalling mechanisms are likely responsible for cell-cell communication has gained momentum of late - namely in the study of extracellular vesicles (EVs), and in particular, exosomes.

Exosomes are vesicles released by a cell which are between 30 and 100nm in diameter, and can be identified by their unique protein and lipid composition, “saucer-like” morphology and expression of exosomal markers including TSG101 and CD63 [145, 146]. Exosomes released from a cell can contain many different components including proteins, lipids, RNA and miRNA [147-150]. Emerging evidence suggests that these vesicles could be, at least in part, responsible for some of the altered characteristics of MSCs found in the TME, and also for some of the tumour promoting effects exerted upon tumour cells by MSCs. For example, Haga *et al.*, found that exposure to tumour cell EVs caused MSCs to express α-SMA, a marker for CAFs, and to secrete CXCL-1, CCL2 and IL-6, factors known to be involved in cancer progression, metastasis and poor prognosis [151-153]. Furthermore, the conditioned medium from MSCs exposed to tumour EVs caused a subsequent increase in tumour cell proliferation and migration [151]. The importance of tumour cell exosomes was evident when Webber *et al.*, noted that exosomes from tumour cells could induce normal prostate stroma to become disease-like in phenotype and function as measured by secretion of soluble factors, angiogenic potential and the ability to promote tumour growth using samples isolated from patients with prostate cancer [154].

**Figure 4:**
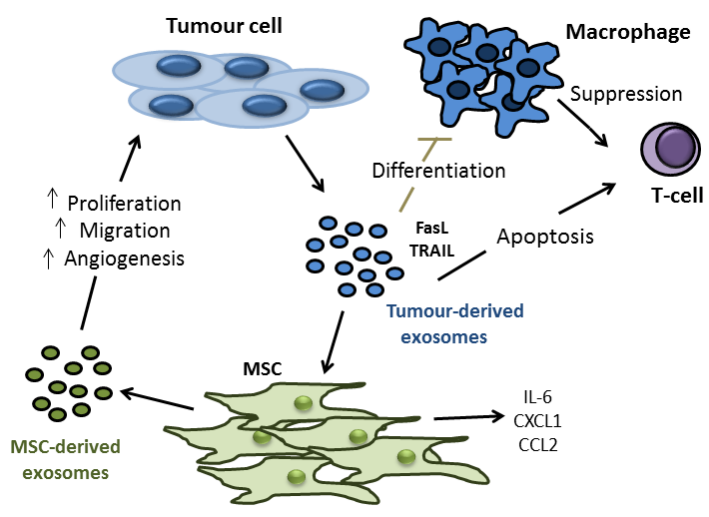
Tumour- and MSC-derived exosomes as potential mediators of colon tumour cell proliferation, migration, angiogenesis and modulation of anti-tumour immune response Experiments involving transwell systems and conditioned medium have shown us that soluble factors released by MSCs can promote all aspects of tumourigenesis including tumour cell proliferation, migration and angiogenesis. Interestingly, more recent experiments involving the treatment of tumour cells with exosomes isolated from MSCs have produced similar results. Exosomes are vesicles released by a cell which are between 30 and 100nm in diameter, making them small enough for easy uptake by target cells. Exosomes released from a cell can contain many different components including proteins, lipids, RNA and miRNA. It is for these reasons that exosomes could represent a key under-explored aspect of tumour-MSC interactions. In addition to MSC derived exosome effects on tumour cells, it has now also been shown that exosomes derived from tumour cells can alter the biology of MSCs, and indeed immune cell components. Tumour derived exosomes can express FasL and TNF-related apoptosis inducing ligand (TRAIL), thus inhibiting macrophage differentiation and inducing t-cell apoptosis. In terms of the effects of tumour released exosomes on MSCs, data to date is limited. Early evidence does show however, that MSCs secrete (IL)-6, chemokine ligand (CXCL)-1 and C-C chemokine receptor (CCR)-2 upon treatment with tumour derived exosomes, all factors which have been implicated in cancer progression, metastasis and poor prognosis.

In addition to exerting direct effects on MSCs, tumour-derived exosomes have also been shown to be capable of modulating the various components of the immune system. With regard specifically to CRC, it has been observed that exosomes secreted from colorectal tumour cells express FasL and TRAIL, thus inducing T-cell apoptosis [155]. Furthermore, it has been shown that colorectal tumour-derived microvesicles impair monocyte differentiation into dendritic cells and those monocytes that do differentiate in the presence of tumour cell microvesicles exerted a suppressive effect on T-cell activity [156].There is also evidence to suggest that exosomes released from MCSs can enhance the growth and angiogenesis of tumours *in vivo*, possibly even to the same extent as MSCs themselves [157] and that these MSC-released exosomes can also induce a state of dormancy in tumour cells that favours their survival until conditions for re-growth and metastasis are optimal [158] (Figure 4). These observed effects allude to a potentially vital role for both tumour- and MSC-derived exosomes in the process of cancer progression, particularly due to their small size and apparent ease of uptake into recipient cells. Therefore, the mediators of exosome uptake may represent novel targets for therapeutic manipulation [159], although a good deal more investigation is needed in this area.

## MSCs IN THE TUMOUR MICROENVIRONMENT - A DOUBLE-EDGED SWORD?

**Figure 5:**
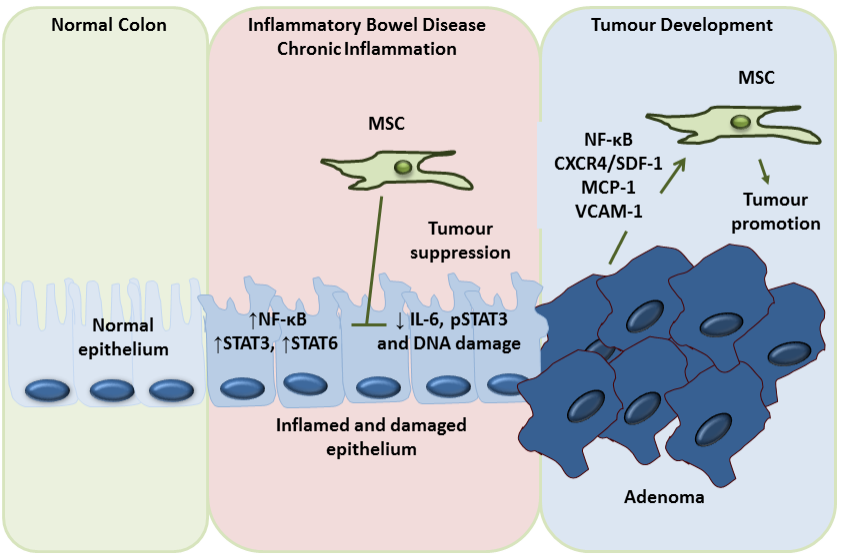
Dual role of MSC in the prevention of inflammation induced colon cancer development and promotion of colon cancer metastasis Although the vast majority of the evidence points to a tumour promoting role for MSCs, there is some evidence to the contrary. However, this anti-tumour effect appears to be specific to the very early stages of tumour development. Throughout the course of a chronic inflammatory condition like inflammatory bowel disease (IBD) the epithelium becomes inflamed and damaged, leading to the production of factors such as nuclear factor kappa B (NF-κB), signal transducer and activator of transcription (STAT) 3 and STAT6, all of which are potentially tumourigenic. It appears that administration of MSCs at this very early stage can have a tumour inhibiting effect by decreasing interleukin (IL)-6 and phosphoSTAT3 signalling and reducing DNA damage. However, once this early stage has passed, MSCs recruited to the tumour by factors such as nuclear factor (NF)-κB, chemokine receptor type 4 (CXCR4), stromal cell derived factor (SDF)1, monocyte chemotactic protein (MCP)-1 and vascular cell adhesion molecule (VCAM)-1 serve only to promote tumourigenesis *via* the mechanisms mentioned throughout this review, namely differentiation to cancer-associated fibroblasts (CAFs), promotion of tumour growth, invasion, metastasis and angiogenesis and the dampening of anti-tumour immunity.

A small number of recent publications add a further level of complexity to the colorectal cancer-MSCs narrative. Studies by Chen *et al.* and Nasumo *et al.*, looked at the impact of MSCs on the development of colon cancer following treatment with azoxymethane (AOM) alone, or in combination with dextran sulphate sodium (DSS) [160, 161]. The DSS-AOM model is a well-accepted model of inflammation-associated colorectal cancer whereby DSS provides the inflammatory insult and is followed by the carcinogen AOM to induce colon tumour formation [162]. In each of these studies, administration of MSCs at different stages of the DSS-AOM protocol resulted in an inhibition of tumourigenesis. Chen *et al.* attributed this reduction to decreased IL-6 and pSTAT3 signalling in colon cells, whereas Nasuno *et al.*, found MSCs prevented initiating cells from sustaining DNA insults and induced G1 arrest in initiated cells *via* TGF-β signalling [160, 161]. Undoubtedly these two studies bring to light some interesting aspects of MSC-colorectal cancer interactions, and raise the question of how MSCs can be anti-tumourigenic in the setting of healthy tissue exposed to a carcinogenic insult, but be pro-tumourigenic once the early tumour initiation phase has passed. Could it be that the MSCs, being immunosuppressive, dampen any inflammatory response that so often leads to tumour development in the colon [163, 164], but that excess anti-inflammatory signalling hinders the tumour-immune response? These models could represent the “elimination” phase of the tumour-immune response mentioned earlier. In the clinical setting colorectal tumours don't usually develop quickly or in response to an acute chemical insult as provided by the DSS-AOM models, and perhaps MSCs are not recruited to the site until later on in the process in contrast to these studies where MSCs were administered in high numbers very early on in the process of tumour development (Figure 5). Further studies refining the precise contributions of MSCs to tumourigenesis at each stage of the cancer development process is vital, particularly when it is noted that MSCs are being investigated for their potential to therapeutically modulate inflammatory diseases of the intestine, including Crohn's disease [165]. These data cited here could also suggest that the role of MSCs in the tumour microenvironment is tumour and context specific, highlighting the urgent need for standardised models.

More recent research has aimed to harness the tumour-homing property of MSCs to deliver cancer-specific drug or gene therapy to patients, with some positive outcomes being observed [166-170]. This concept arose from MSCs remarkable ability to ‘home’ to tumours. In fact the first clinical trial using MSCs as an anti-cancer therapy is currently underway in Germany (eudract_number:2012-003741-15). Although no results have been published to date, this is yet another interesting facet of the MSC-tumour story where the immunological consequences of MSCs may have more important effects. The rationale behind this study is to engineer a patient's own MSCs to express therapeutic gene of interest and make use of the tumour-homing capacity of MSCs so as to allow for IV administration of the engineered MSCs [171] These MSCs will, however, also be transfected with a replication-incompetent and self-inactivating vector system so as to avoid any potential adverse effects of a bolus of MSCs being recruited to the TME, one of which effects would presumably include immunosuppression and potential tumour-immune evasion. [171]. This trial is based upon a number of pre-clinical studies showing potential efficacy in inhibiting tumour growth or metastasis of MSCs engineered to express anti-cancer therapies such as CX3CL-1, TRAIL and IFN-β [169, 172-174]. However, as with all pre-clinical studies, these results were obtained over a short period of time in animals with exogenously administered tumours displaying a much accelerated growth rate compared to that of patients. For these reasons, and the fact that the follow up periods in these studies were short, and thus limited the analysis of long term immunological consequences, these results must be interpreted with caution.

A final important aspect of CRC-MSC interactions is the potential impact that these cells could have on colorectal cancer therapy. Recent research has highlighted the ability of MSCs to contribute to resistance to chemotherapeutics in both haematological malignancies and solid tumours [175-177]. In CRC 5-Fluorouracil (5-FU) is one of the first-line therapies for metastatic disease [178]. However, it has now been shown that a population of bone-marrow MSCs exist that are both potently immunosuppressive and 5-FU resistant, a dangerous combination in the context of CRC chemotherapy [179]. Difficulties also arise with the use of radiotherapy [180]. MSCs have been shown to be radio-resistant [181, 182] and so could withstand such therapy and retain their tumour-conditioned phenotype, thus potentially contributing to disease relapse.

**Table 2:** Key under-explored areas of research into the effects exerted by stromal cells in the tumour microenvironment

**Key under-explored areas of research into the effects exerted by MSCs in the tumour microenvironment (TME)** The identification of definitive markers of cancer-associated fibroblasts Precise mechanisms by which MSCs are recruited to the TME The exact ratio of MSCs present in the TME at various stages of cancer progression The factors responsible for MSC-mediated M2 polarisation of macrophages The factors that allow MSCs supress innate immune functions The mechanisms by which MSCs suppress T cell proliferation and effector functions The ability of MSCs to behave as immune cells and the consequences of this for tumour growth and progression The role played by both tumour- and MSC-derived exosomes in tumour progression The influence of MSCs on chemo- redio- and immuno-therapy resistance in colon tumours

## FUTURE PERSPECTIVES

Following review of recent publications as detailed here, it is clear that the impact of tumour-educated MSCs on the immune system plays a significant role in MSC-mediated tumour promotion. The putative mechanisms of this element of assisted immune escape are elucidated, albeit with varied results as to particular mechanisms responsible. Nevertheless, key areas in which gaps remain in our knowledge include the precise mechanisms by which tumours recruit MSCs, or activate tissue resident MSCs, the identification of specific markers to reliably identify and quantify MSCs in the tumour microenvironment, the precise mechanisms by which tumour-educated MSCs influence immune cell components and the mechanism of release, uptake and consequent signalling induced by extracellular vesicles by both MSCs and tumour cells, not to mention determining the actual content of these vesicles.

Furthermore, as was noted by Khong and Restifo in their review of tumour-escape phenotypes in 2002 [183], all of these results must be interpreted with caution. These studies are carried out in mice that have been administered a high dose of potent, fast growing, often allogeneic, tumour cells, and very often a similarly high dose of MSCs, and so the results from these studies cannot be interpreted as accurately representing what is often the very slow and poorly understood process of cancer development in humans. These words of caution point to an ever increasing need for more relevant pre-clinical models of spontaneous tumour development in future studies. Together, with more sensitive imaging systems, the role of MSCs in spontaneous tumour development, will certainly unfold in the near future. Until these questions are answered and data is available from human specimens, the use of MSCs as a cellular therapy for patients with a genetic predisposition or those at an inherent increased risk for tumour development must be translated with great caution. Finally, to date, no therapies routinely used in colorectal cancer treatment specifically target stromal cell mediated immunomodulation. A better understanding of the albeit complex interactions underpinning these effects and a broadening of our understanding of how to identify these cells and characterise their interactions with other components of the TME will undoubtedly lead to the development of more targeted and efficacious anti-cancer therapeutics.
